# Analysis of Hop Stunt Viroid Diversity in Grapevine (*Vitis vinifera* L.) in Slovakia: Coexistence of Two Particular Genetic Groups

**DOI:** 10.3390/pathogens12020205

**Published:** 2023-01-28

**Authors:** Peter Alaxin, Lukáš Predajňa, Adam Achs, Zdeno Šubr, Michaela Mrkvová, Miroslav Glasa

**Affiliations:** 1 Institute of Virology, Biomedical Research Center of Slovak Academy of Sciences, Dúbravská cesta 9, 845 05 Bratislava, Slovakia; 2 Department of Biology, Faculty of Natural Sciences, University of Ss. Cyril and Methodius in Trnava, Námestie J. Herdu 2, 917 01 Trnava, Slovakia; 3 Department of Molecular Biology, Faculty of Natural Sciences, Comenius University in Bratislava, Ilkovičova 6, 842 15 Bratislava, Slovakia

**Keywords:** grapevine, high-throughput sequencing, HSVd, mixed infection, RNA secondary structure, viroid

## Abstract

The hop stunt viroid (HSVd) is a widespread subviral pathogen infecting a broad spectrum of plant hosts including grapevine (*Vitis vinifera* L.). Despite its omnipresence in virtually all grapevine growing areas around the world, molecular data characterizing HSVd populations are missing from Slovakia. Analysis of the complete nucleotide sequences of 19 grapevine variants revealed the existence of two genetic HSVd groups in Slovakia (internally named the “6A” and “7A” groups based on the particular stretch of adenines at nucleotide positions 39–44/45, respectively). Despite their sampling at different times in various unrelated vineyards, the 6A and 7A groups are characterized by low intra-group divergence (~0.3 and 0.2%, respectively). On the other hand, inter-group divergence reached 2.2% due to several mutations, seven of which were found to be group-specific and mainly (except for one) located in the region of the pathogenic domain. Interestingly, in addition to their frequent co-existence within the same geographical location, the mixed infection of the 6A and 7A type sequence variants was also unequivocally and repeatedly proven within single grapevine plants. The RNA secondary structure analysis of representative isolates from each of these two genetic groups indicated a potential compensatory explanation of such mutations. These group-specific sites could be pointing towards the evolutionary selection linked to the necessity of the viroid to retain its structural conformational integrity, crucial for its functional biochemical ability to interact with specific grapevine cellular host factors required for HSVd propagation.

## 1. Introduction

Viroids represent a small (regarding the overall number of species [1]) but very widespread and successful group of phytopathogens, known only from higher plants, characterized by a very simple molecular composition. Categorized as subviral infectious agents, their whole structure is constituted of only one single-stranded RNA molecule, with covalently bound ends forming a circle [2,3]. Viroid “genomes” do not code for any gene [4], and thus are lacking any of their own protein or lipid components. Despite this fact, these infectious RNA molecules can still have a significant impact on overall plant physiology. Compared to their structurally more complex viral counterparts, viroids can induce a wide range of symptoms on plants, often resulting in important agronomical losses reported from different countries around the world [5,6,7,8,9]. Viroids owe their remarkable biochemical activity to the secondary structure of their RNA molecules (forming even three-dimensional functional motifs [10]), which are folded because of intramolecular base-pairing (conventional Watson–Crick as well as wobble). The resulting conformation thus basically functions as double-stranded RNA, which is a hypothesized model for viroids’ ability to hijack eucaryotic cellular proteins (some of which even normally do not recognize RNA as their substrate) in order to exploit them for their own replication and cell-to-cell transport [11,12,13,14].

The hop stunt viroid (HSVd, a type species of the genus Hostuviroid) belongs to the *Pospiviroidae* family, the larger one of the two viroid families recognized to date [15]. A typical characteristic of *Pospiviroidae* members is the localization in the cellular nucleus regarding their replication strategy. This is in contrast with the chloroplast-targeting and even own-ribozymal activity possessed in the family *Avsunviroidae* [1,3,15]. The HSVd has a broad host range, thus posing as a potential threat to agriculture in the world. Its first description was triggered by the study of the causal agent of shoot growth dwarfism (stunt symptom) in hop (*Humulus lupulus* L.; *Cannabaceae*) in Japan [16,17]. The HSVd also includes four other sequence variants from mutually different hosts, named after their host-specific symptomatology, i.e., the cucumber pale fruit viroid (CPFVd; previously regarded as a separate species [18,19]); the citrus cachexia viroid (CCaVd, the causal agent of the so-called cachexia xyloporosis disease of citrus trees [20,21,22,23]); the dapple peach (fruit disease) viroid, and the dapple plum (fruit disease) viroid in *Prunus* spp. [24,25,26,27]. Moreover, several other agriculturally and economically important plants can also be infected with HSVds, e.g., apricot, almond (*Rosacea*), fig, mulberry (*Moraceae*), pistachio (*Anacardiaceae*), and kumquat (*Rutaceae*) [27,28,29,30]. However, in many cases, the HSVd infection can stay asymptomatic [22,27].

Grapevine (*Vitis vinifera* L.; *Vitaceae*) is another well-known, widespread, and often symptomless natural HSVd host [31,32]. Nonetheless, the research on viroids in grapevines in Slovakia has previously received little attention [33]. As for other pathogens, the diversity of HSVd populations plays a significant role in the epidemiology and etiology of the viroid disease, and its understanding constitutes an important prerequisite to develop accurate diagnostics and efficient control strategies. Therefore, in this work, standard and high-throughput sequencing (HTS) tools were used to analyze HSVd molecular variability in Slovakia, leading to the identification of two grapevine-specific molecular groups of variants, acting often in mixed infections.

## 2. Materials and Methods

### 2.1. Grapevine Samples

The HSVd variants characterized in this work were obtained from grapevine plants (*V. vinifera* L.) sampled in different unrelated vineyards and from different cultivars from 2012 to 2021 as a part of the effort to monitor the health status of grapevines in Slovakia (Table 1). Targeted plants were selected randomly with no specific relation to plant symptomatology. Total RNAs were then extracted either from cortical scrapings of one-year-old canes (when collected in the dormant period) or leaf petioles (from leaves collected during the vegetation period) using a Spectrum^TM^ Plant Total RNA Kit (Sigma-Aldrich, St. Louis, MO, USA) and stored at −80 °C prior to further analysis.

### 2.2. High-Throughput Sequencing and Analysis

Ribosomal-RNA-depleted preparations (Ribo-Zero rRNA Removal Kit, Illumina, San Diego, CA, USA) from three grapevine samples (SK30, SK503, and SK933; Table 1) were used for double-stranded cDNA synthesis using a SuperScript II Kit (Thermo Fisher Scientific, Waltham, MA, USA). The samples were further processed with a Transposon-based Chemistry Library Preparation Kit (Nextera XT, Illumina, San Diego, CA, USA) and the cDNA library was sequenced on the Illumina MiSeq platform (Illumina, San Diego, CA, USA; 300-bp paired-end sequencing) as previously described [34,35]. From 1.1 to 2.4 million high-quality reads per sample were used for de novo assembling using CLC Genomics Workbench 7.5 and Geneious v. 8.1.9 software. The obtained contigs were blasted to the viral/viroid genome database (ftp://ftp.ncbi.nih.gov/genomes/Viruses/all.fna.tar.gz). The complete nucleotide sequences of the HSVd genomes obtained from HTS data reported herein were deposited into the GenBank database under accession numbers MN548397, OP918897, and OP918898.

### 2.3. RT-PCR Detection, Sanger Sequencing, and Phylogenetic Analysis

Additional total RNAs isolated from grapevine samples were used as templates for a two-step RT-PCR. The first-strand cDNA was synthesized with the reverse transcription of total RNA using hexameric random DNA primers and an AMV Reverse Transcriptase (both from Promega, Madison, WI, USA). An aliquot of cDNA was then used as a template in a standard PCR for the complete viroid sequence amplification using TaKaRa Ex Taq polymerase (Takara Bio Inc., Shiga, Japan) and the HSVd-specific primer pair HSV-78P (5′ aaCCCGGGGCAACTCTTCTC-3′; sense) and HSV-83M (5′ aaCCCGGGGCTCCTTTCTCA-3′; antisense, lowercase letters represent the non-viroid sequence) partially overlapping in the central conserved region (CCR) of the HSVd [31]. The PCR cycling conditions were as follows: initial denaturation 95 °C for 2 min; 40 cycles (denaturation at 95 °C for 30 s; annealing at 57 °C for 30 s; elongation at 72 °C for 20 s); and final elongation at 72 °C for 5 min. Amplified PCR fragments were then analyzed by the standard agarose gel electrophoresis on 5% agarose gel (UltraPureTM Agarose-1000; Thermo Fisher Scientific, Waltham, MA, USA). 

Selected amplicons were subsequently purified from the gel using Wizard^®^ SV Gel and PCR Clean-Up System kits (Promega, Madison, WI, USA). Purified PCR products were subjected to Sanger sequencing (Eurofins Genomics, Vienna, Austria) primed by the same HSVd-specific primers used for RT-PCR. To study the intra-isolate variability, two purified PCR products were cloned into pGEM^®^-T Easy Vector (Promega, Madison, WI, USA) and the individual clones (6 clones per amplicon) were bidirectionally Sanger sequenced using pUC/M13 primers (Promega, Madison, WI, USA). The obtained HSVd complete sequences were deposited into the GenBank database under accession numbers OP918899–OP918914 (Table 1).

The genomic sequences obtained in this work, together with the representative HSVd sequences retrieved from the public database (https://www.ncbi.nlm.nih.gov/nucleotide/) were analyzed using the Molecular Evolution Genetics Analysis (MEGA7) program [36], using the p-distance model.

The prediction of RNA secondary structures of the selected variants was performed using the online web tool Mfold (available at http://www.unafold.org/mfold/applications/rna-folding-form.php) [37], with the default parameters for circular RNAs.

## 3. Results and Discussion

### 3.1. Identification of HSVd in the Grapevine Virome

The previous HTS analyses of three grapevine samples (SK30, SK503, and SK933) revealed a complex infection of plants by several viruses, i.e., grapevine leafroll-associated virus-2; grapevine leafroll-associated virus-3 (GLRaV-3); grapevine rupestris stem pitting-associated virus (GRSPaV); grapevine rupestris vein feathering virus (GRVFV); and grapevine Syrah virus-1 (GSyV-1) [38,39,40]. In addition to these several viral pathogens, the HSVd was also identified to be present in the samples [33,40]. In all three samples, the obtained HTS data enabled the reconstruction of the complete genomic sequences of HSVd with high average coverage (17.1–395.4x). Interestingly, although originating from unrelated grapevines sampled over a five-year period, the three HSVd genomes were strictly collinear (297 nts long) and 100% identical. High sequence similarity (identical for some variants even across the time and geographical distribution) can be similarly observed among database grapevine HSVd sequences [32]. Such sequence rigidity could be a consequence of the antiquity of the HSVd pathogen in grapevines as an infectious RNA [31,32] and the vegetative propagation of grapevines and their distribution around the world.

To further assess the occurrence of HSVd in Slovak grapevines (not studied previously), an additional 52 grapevine samples from different vineyards were analyzed with RT-PCR [31], from which 38 in total tested PCR-positive (the HSVd prevalence in tested samples reached 73.08%). This suggested the ubiquitous presence of this viroid species in Slovak vineyards.

### 3.2. Identification of Two Molecularly Different HSVd Groups

Out of 38 HSVd-positive samples, we further analyzed 14 samples, selected to ensure their diversity based on the vineyard location or cultivar differences (Table 1). From 12 samples, the complete HSVd genomes were obtained with the direct dideoxy Sanger sequencing of amplicons. Contrary to previous HTS-based reconstructed HSVd genomes, the sequences of 12 of these HSVd genomes (OP918899–OP918910) showed a clear length dichotomy with the occurrence of 297 nucleotides, as well as 298-nucleotide-long variants. The multiple alignments of Slovak HSVd genomes confirmed their splitting into two genetically different molecular groups, characterized by a low mean nucleotide intragroup divergence (reaching 0.3% and 0.2%), while the inter-group divergence reached 2.2%.

**Figure 1:**
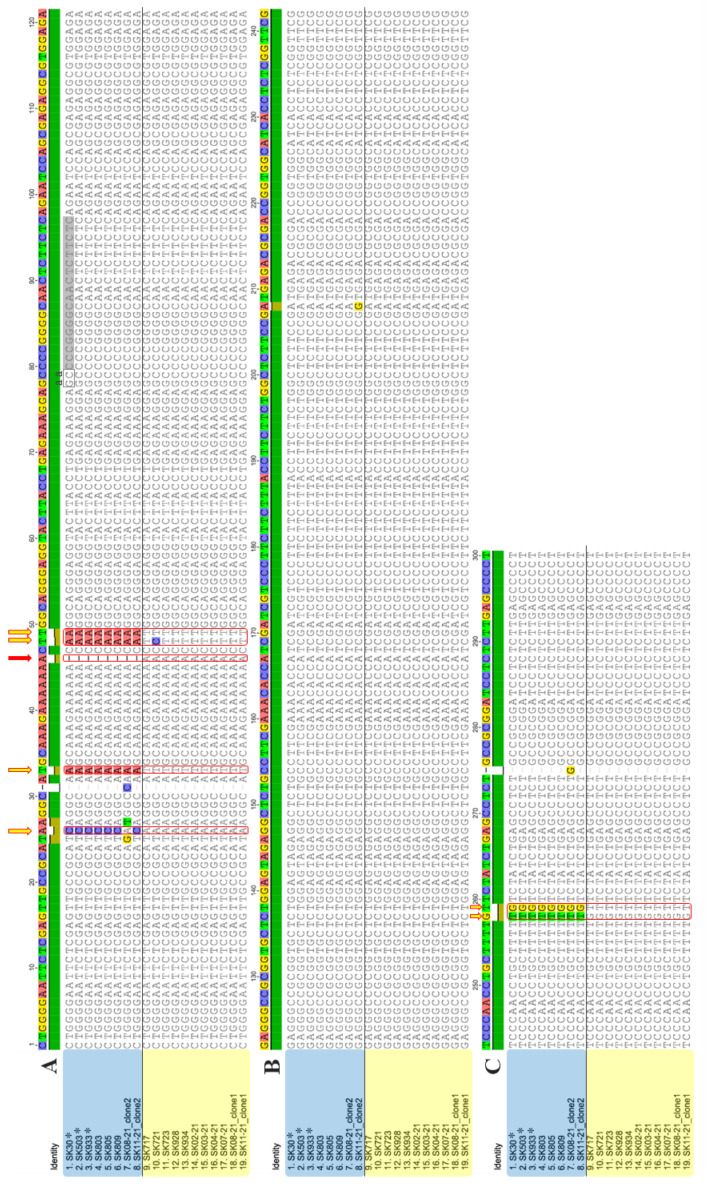
The alignment of 19 whole-viroid sequences of HSVds from 17 tested grapevines from Slovakia. (**A**,**C**) the positions of the crucial and cluster-dividing mutations spread across the pathogenicity and terminal left domains (yellow arrows and rectangles with red outliners) with the one (red arrow) possibly initiating the emergence of all of the others. Other colored bases represent the positions of only one-variant-specific single-nucleotide mutations. (**A**,**B**) the region (representing the central, variability, and terminal right domains) that was uniformly conserved among all 19 Slovak grapevine isolates (from positions 100 to 200, approximately). Isolates belonging to the 6A cluster are in blue rectangles and isolates belonging to the 7A cluster are in yellow rectangles. The three sequences determined from the HTS data are marked by asterisks. The sequence of the forward primer HSV-78P [31] is marked by grey rectangles.

**Figure 2:**
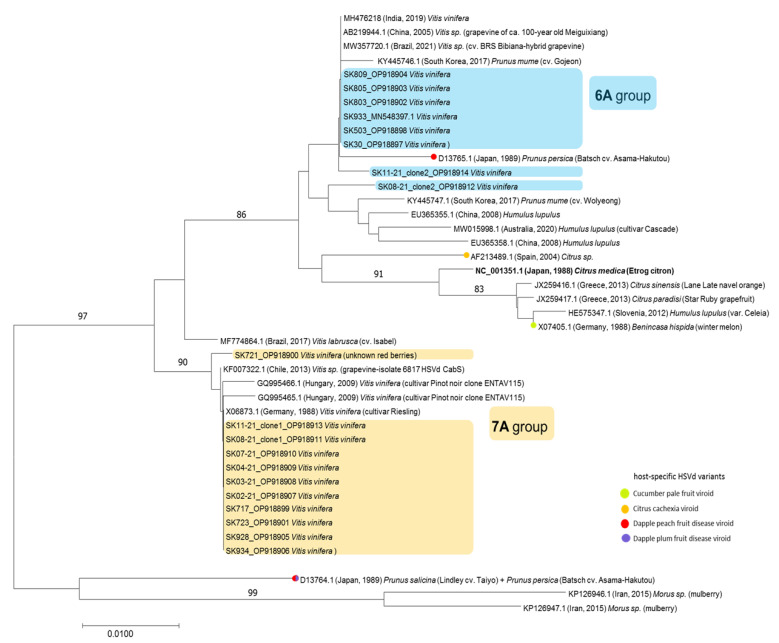
Neighbor-joining phylogenetic tree constructed in MEGA7 from alignments of 19 complete HSVd sequences generated in this study and 23 database sequences. Slovak HSVd isolates are in blue or yellow rectangles, depending on their grouping. The database sequences are identified by their accession numbers, host, and geographical location. Only bootstrap values ≥ 70% (1000 bootstrap re-samplings) are indicated. The scale bar indicates a genetic distance of 0.01.

The first group (consisting of 3 isolates) was characterized by a genome 297 nt in length and a typical stretch of 6 adenosines at positions 39–44 (referred to here as the 6A group, Figure 1 and Figure 2). On the other hand, 9 isolates formed the second group, with a genome consisting of 298 nt and characterized by 7 adenosines at positions 39–45 (referred to here as the 7A group). Interestingly, one of the adenosines at positions 39–45/46 was responsible for the 1 nt difference in length between the two genetic groups. 

In addition to the different adenosine stretches, the alignment revealed a total of seven mutations located in the positions spanning the pathogenic and terminal left domains, having a clear group-specific character (Figure 1). This grouping was also confirmed by phylogenetic analyses (Figure 2). However, due to the mixed infection of grapevines involving HSVd and other viral pathogens mentioned above, the contribution of the two HSVd groups to symptomatology (if any) in grapevine could not be determined. The fact that HSVds are reported mostly asymptomatically in grapevine (in contrast to in hop) could be linked to mutual long-term adaptation and the assumption that the HSVd pathogen originates from grapevine as its natural source [31].

### 3.3. Mixed Infection of HSVd Variants

Although in most of the cases, direct dideoxy Sanger sequencing resulted in unequivocal sequence reads, we were not able to obtain clean whole-genome HSVd sequences from 2 of 14 samples. The visual inspection of chromatograms from directly sequenced amplicons of the SK08-21 and SK11-21 samples showed an occurrence of several mixed signals, interestingly, mainly in the same 7 positions responsible for HSVd grouping in the previously characterized 12 samples (Supplementary Figure S1). To elucidate this problem, we cloned the PCR amplicons and sequenced six individual clones per amplicon. As expected, two distinct sequence variants belonging to the 6A and 7A groups were obtained from each of these two samples (OP918911 and OP918913 for SK08-21, and OP918912 and OP918914 for SK11-21) (Figure 1). For both samples, the ratio between the 7A and 6A group variants was 2:1. 

The identification of the HSVd-variant mixed infection in single grapevine plants prompted us to re-analyze the HTS datasets of the SK30, SK503, and SK933 samples, from which the 6A group variants were determined by default Geneious mapping (MN548397, OP918897, and OP918898). Interestingly, by remapping the datasets to represent the 6A and 7A sequence variants (SK11-21_clone1 and SK11-21_clone2, respectively) using strict parameters (Geneious mapper, identity 99%, overlap 20–30 bp), we found a mix of both types of groups in the SK503 and SK933 samples. For example, the coverage of the two variants identified in the SK503 dataset was 116.0x for the 6A group variant and 63.0x for the 7A group variant. Moreover, the visual inspection of mapping confirmed the presence of reads representing both group variants, containing the same seven group-dividing positions. These results confirmed and further pinpointed the complex and heterologous nature of viroid populations in some grapevine samples, already noted for grapevine viruses [39]. 

Because of the sole-6A-variant character of SK30 and the “prevailing” 6A group variant in the SK503 and SK933 samples, only those sequences were added to the final alignment. Moreover, due to the mutual shared nucleotide identity across the extensive parts of the HSVd genome (Figure 1A,B), the major quantity of 7A-like reads would have been unified and indistinguishably mixed with the identical 6A-like reads. The overall actual ratio between the 6A and 7A groups was thus very difficult to determine from the HTS datasets. Nonetheless, it would be interesting to also study the prevalence of these two groups in grapevine plants, especially in conjunction with the observations of whether one of the groups is more abundant (more copies of genomic RNA) in this host or if their numbers undergo some sort of oscillation across time.

Our molecular data fit with previously published results of molecular diversity in conjunction with different host adaptations performed in Japan [32]. In their study, natural HSVd-grapevine variations resulted in the original observation of the exact same seven mutated positions. Interestingly, in this study, the most abundant mutations were those belonging to the 6A group, contrary to the larger prevalence of the 7A group variants in Slovakia.

To further evaluate the significance of the 6A- and 7A-group-belonging mutations, we also analyzed all available grapevine HSVd genomes retrieved from the GenBank database ((n = 228), accessed on 1 October 2022). The robust alignment data again pinpointed the dichotomic nature of the 7A-group-specific mutations (i.e., occurring only as 6A- or 7A-specific ones, without random or uncorrelated mutations, Table 2). Similar to Kawaguchi-Ito et al. [32], the 6A group variants were found to be more abundant within the database. It should be noted, however, that some grapevine HSVd isolates displayed particular patterns of mutations in these parts of the genome, which did not support the clear-cut division [41] observed in the Slovak samples. This indicated the long-term diversification of the HSVd in grapevine hosts and the possible role of non-host selection pressures. 

### 3.4. Group-Defining Mutations vs. RNA Secondary Structure

Previous sequence analyses led us to define the seven specific mutations located in the positions spanning the pathogenic and terminal left domains as the common divergence factor within the Slovak HSVd variants (Figure 1). Taking also into consideration the overall low molecular variability observed in the remaining parts of the HSVd genome in both groups, we analyzed the relationship between these mutations and predicted an RNA secondary structure. We based our approach on the necessity of the viroid molecule to maintain its secondary RNA structure in order to preserve its biological functionality [11,12,42]. 

Therefore, two representative genomes from each genetic group (SK805 (OP918903) from the 6A group and SK723 (OP918901) from the 7A group) were subjected to the secondary structure analysis. 

**Figure 3:**
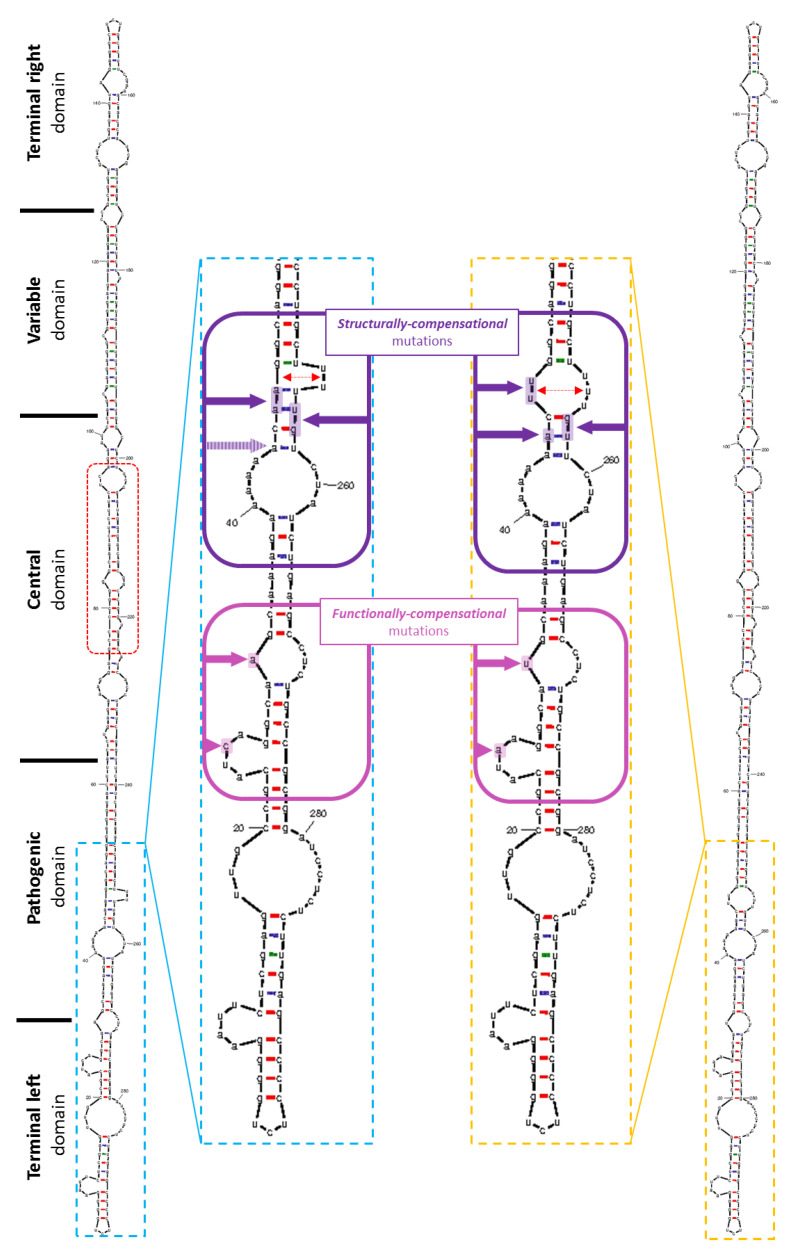
The secondary structure of two representative complete HSVd sequences, i.e., SK805 (6A group, blue color) and SK723 (7A group, yellow color). The Gibbs free energy for the most thermodynamically stable conformation, calculated via Mfold software [37], was −114.0 and −114.10, respectively. The central conserved region (CCR), into which the diagnostic primers were targeted, is marked (red rectangle). The speculated compensational mutations were spread only across the terminal left and pathogenic domains. Each mutated base in both isolates are marked by arrows (purple for structurally (also indicated by double red arrow) and pink for functionally considered mutations).

As previously predicted for all members of the *Pospiviroidae* family [1,3,43], a typical rod-like shape secondary structure was obtained after folding [37] for both genome types (Figure 3). Nevertheless, the secondary structure analysis of the two HSVd variants provided a potential explanation for the seven-group-forming mutated positions. Hypothetically, those could have started as compensations for the nucleotide-base-length shift, probably triggered by the one specific adenosine insertion in positions 44/45 (Figure 1) due to the eucaryotic-DNA-dependent RNA II polymerase “slip” and base addition when replicating poly(N) sequences [44,45,46]. One can hypothesize that this could be the first candidate for the mutation having caused the splitting of these two sequence groups and triggered subsequent compensatory mutations in the HSVd-grapevine population in order to maintain the infectious functionality of their RNA secondary structures. The hypothesis of a six-adenosine stretch being the original variant is seemingly also supported by the previously mentioned study of HSVd-grapevine variants in Japan [32]. Finally, our samples SK30 (OP918897); SK503 (OP918898); SK933 (MN548397); SK803 (OP918902); SK805 (OP918903); and SK809 (OP918904) from the 6A group were also 100% identical to the AB219944 genome [31] obtained from 100-year-old grapevine with the corresponding length of 297 nucleotides.

Indeed, when observing the positions 256–257 (257–258 in the 7A group), we noted the exchange of order from U–G (6A group) to G–U (7A group), probably due to the necessity of base-pairing with the additional A in position 45 for the 7A group (Figure 3). The inserted A would have caused the next-in-line C (position 46 in SK723) to be opposite to U instead of G (positions 256 and 257, respectively, as it is in SK805). Such an exchange from U–G (256–257; 6A group) to G–U (257–258; 7A group), observable in our data, would be a very simple and effective way to deal with such a length-changing mutation. Additionally, the exchange of A–A in positions 46–47 (6A group) for U–U in positions 47–48 in the 7A group could also be an explanation for the maintenance of structural integrity. This exchange seemed to stabilize the loop consisting of two uracils in the 6A group, which was formed only on the lower strand. In the 7A group, this loop was formed on both strands and consisted of five uracils (Figure 3). Yet again, one adenosine insertion would have caused these two uracils (positions 253–254; 6A group) to be in opposition to the two adenosines (A–A in mutated positions 46–47; 6A group), which would have led to intra-molecular base-pairing resulting in potential loop closing. The presence of such loop motifs in the secondary structure of RNA is crucial for viroid replication and trafficking in host cells, described mainly in Potato spindle tuber viroid (PSTVd) species [11,14,47]. Interestingly, in the case of the PSTVd, the periphery of its pathogenic domain contains motifs responsible for supposed interactions with cellular proteins involved in viroid trafficking [11,47], which could also be the case in HSVd populations. This discussed loop is, although larger, still preserved in the 7A group due to the substitutions exchanging A–A to U–U (in positions 47–48). Therefore, we denoted all these four positions (plus the above-mentioned A insertion) as they contained the potentially structurally compensational mutations due to the one-adenosine insertion. Similar compensation was also observed in the case of the two unique insertions of the variant SK08-21_clone2 (OP918912) (additional C in position 31 was stabilized via base pairing with an additional G in positions 274/275) (Supplementary Figure S2), again supporting such a type of mechanism for viroid RNA adaptability when dealing with random nucleotide insertions.

The remaining two group-specific mutated positions were apparently without any structural consequences, as both loops they were mapped into had in both groups the same structural integrity (Figure 3). They included an exchange of C (6A group) to A (7A group) in position 26 and the exchange of A (6A group) to U (7A group) in position 32. Because of the lack of structural interpretation, they could potentially have some functionally important compensatory meaning. For example, in the PSTVd, the very beginning of the RNA sequence (the terminal left domain) is crucial for replication, as it contains just those motifs recognized by RNA Pol II as well as by the unique splicing form of transcription factor IIIA (TFIIIA-7ZF) [11,13,14,47]. Experimental data regarding HSVd infectivity and, more importantly, symptom severity in cucumber (*Cucumis sativus* L.) [48], provided us with another potential clue for their interpretation. 

Moreover, Xia et al. [48] used two HSVd variants (hop and grapevine) to study the involvement of mutations to symptomatology in cucumber. Interestingly, from 4 mutually differing positions, one mutation was located at position 26. The grapevine variant, causing severe symptoms in cucumber, had C in this position, similar to the 6A group. On the other hand, the hop variant, causing only mild symptoms, had A in position 26, similar to the 7A group. 

## 4. Conclusions

The importance of studying the exact points and changes in the nucleotide sequence of the viroids for epidemiology purposes was supported by previous ecological as well as experimental studies. Those range from new host adaptations (e.g., the PSTVd on *Nicotiana tabacum* via a single-nucleotide substitution in the lower strand of the CCR [49]) to symptom expression (e.g., in the case of the HSVd, only a few, even if single nucleotide changes in the variable domain, can alter the severity of citrus cachexia symptoms [50]). This simple mutation dependence reflected two intertwining molecular principles: (i) the functional simplicity of viroids (RNA sequence) interacting with functionally similar cellular proteins of (ii) slightly different substrate specificities in different plant hosts due to their mutual structural and, more importantly, conformational (subtle) changes [1].

In this paper, we report the analysis of the complete genomic sequences of 19 HSVd-grapevine variants, which led to the finding of two specific-mutation-dependent molecular groups spread throughout Slovakia. It is noteworthy that these same specific mutations were previously reported by Kawaguchi-Ito et al. [32]. This fact ought to be explained by two factors: first, the antiquity of the HSVd pathogen as an infectious RNA molecule and its necessity to maintain its secondary RNA structure. To preserve its biological functionality, only a small number of mutations can be tolerated, and, at the same time, only in the specific locations of the RNA molecule. The second is the vegetative propagation of *V. vinifera* cultivars around the world, mainly for agricultural and breeding purposes. Moreover, grapevine, as the hypothesized original source of the HSVd [31], can create the specific host intracellular evolutionary pressure via its specific viroid-exploited enzymes.

Although the importance of group-specific mutations highlighted in this work was strongly supported by in silico data, their biological significance and correlation to HSVd infectivity and/or symptom severity must be experimentally tested. Such an understanding of the diversity of grapevine HSVd populations would have a significant role in the epidemiology of the viroid and constitutes an important prerequisite for the development of accurate diagnostics and efficient control strategies.

## Figures and Tables

**Table 1:** List of Slovak HSVd isolates with some characteristics.

Sample	Host	Locality	Year of Sampling	GenBank Accession Number
SK30 *	*V. vinifera*, cv. Veltliner	Pezinok	2012	OP918897
SK503 *	*V. vinifera*, cv. Dornfelder	Šenkvice	2015	OP918898
SK933 *	*V. vinifera*, unknown white berries	Pezinok	2017	MN548397
SK717	*V. vinifera*, unknown white berries	Pezinok	2016	OP918899
SK721	*V. vinifera*, unknown red berries	Pezinok	2016	OP918900
SK723	*V. vinifera*, cv. Muller-Thurgau	Pezinok	2016	OP918901
SK803	*V. vinifera*, cv. Palava	Pezinok	2017	OP918902
SK805	*V. vinifera*, cv. Veltliner	Pezinok	2017	OP918903
SK809	*V. vinifera*, cv. Welshriesling	Pezinok	2017	OP918904
SK928	*V. vinifera*, cv. Silvaner	Limbach	2017	OP918905
SK934	*V. vinifera*, cv. Dornfelder	Cífer	2017	OP918906
SK02-21	*V. vinifera*, unknown red berries	Pezinok	2021	OP918907
SK03-21	*V. vinifera*, unknown red berries	Pezinok	2021	OP918908
SK04-21	*V. vinifera*, cv. Traminer	Pezinok	2021	OP918909
SK07-21	*V. vinifera*, unknown white berries	Pezinok	2021	OP918910
SK08-21	*V. vinifera*, cv. Veltliner	Pezinok	2021	OP918911 (clone 1) OP918912 (clone 2)
SK11-21	*V. vinifera*, cv. Muller-Thurgau	Pezinok	2021	OP918913 (clone 1) OP918914 (clone 2)

* Whole viroid sequences generated from the HTS data.

**Table 2:** Frequency of the occurrence of seven group-defining mutations in complete HSVd sequences from grapevine available in GenBank. The total number of analyzed sequences was 228; however, some contain degenerated bases in the analyzed positions; thus, the overall sum of the counted positive sequences is >228 in each row (for clarity, the data are also converted to percentage).

Nucleotide Position *	6A Group Nucleotide/Number of Sequences/%	7A Group Nucleotide/Number of Sequences/%
26	C/151/62.66	A/90/37.34
32	A/156/64.20	U/85/35.00 **
44/45	Δ/158/65.56	A/83/34.44
46/47	A/162/66.94	U/80/33.06
47/48	A/185/77.40	U/53/22.18 ***
256/257	U/170/70.83	G/70/29.17
257/258	G/166/69.17	U/74/30.83

* Listed according to SK805_OP918903/SK723_OP918901; ** 2 out of 228 references (0.80%) had a different base in this position (G); *** 1 out of 228 references (0.42%) had a different base in this position (C); Δ: nucleotide deletion.

## Data Availability

The nucleotide sequences reported in this paper are deposited into the GenBank database (www.ncbi.nlm.nih.gov) under the accession numbers listed in the text.

## Supplementary Materials

The following supporting information can be downloaded at: https://www.mdpi.com/article/10.3390/pathogens12020205/s1, Figure S1: chromatogram of the PCR amplicon of the SK11-21 sample obtained by Sanger sequencing indicative of mixed infection by different HSVd variants; Figure S2: secondary structure of the SK08-21_clone2 (6A group).
