# Supplementary material for: Analysis of Hop Stunt Viroid Diversity in Grapevine (Vitis vinifera L.) in Slovakia: Coexistence of Two Particular Genetic Groups
Source: Pathogens. 2023 Jan 28;12(2):205. doi: 10.3390/pathogens12020205 (PMC9965860; doi:10.3390/pathogens12020205)
Supplement: Supplementary file 1 [file pathogens-12-00205-s001.zip › Supplementary Figure S2_Alaxin_rev1.pptx]

## Slide 1
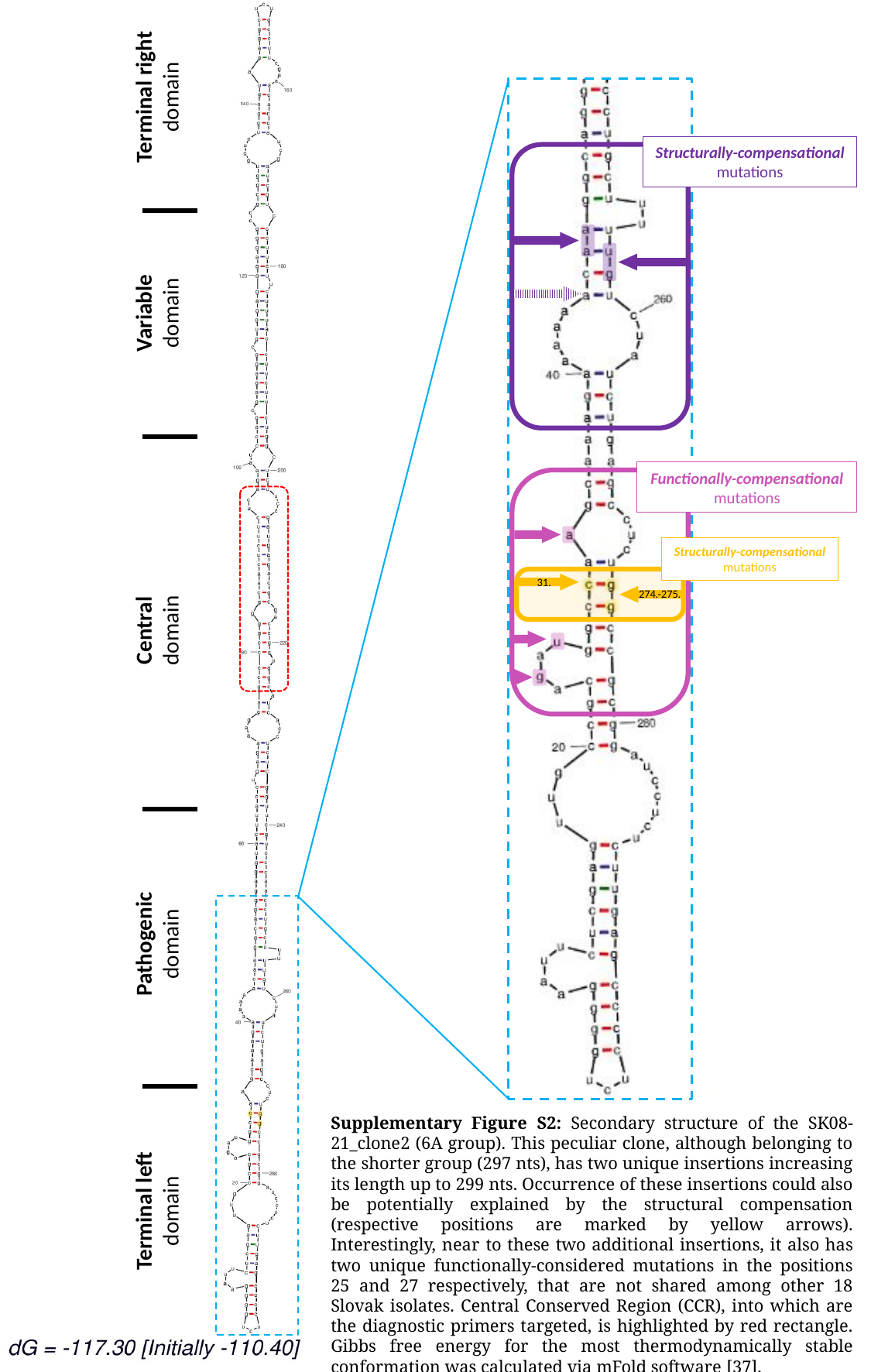

Terminal right
domain
Variable
domain
Central
domain
Pathogenic
domain
Terminal left
domain
31.
274.-275.
Structurally-compensational
mutations
Functionally-compensational
mutations
274.-275.
Structurally-compensational
mutations
Supplementary Figure S2: Secondary structure of the SK08-21_clone2 (6A group). This peculiar clone, although belonging to the shorter group (297 nts), has two unique insertions increasing its length up to 299 nts. Occurrence of these insertions could also be potentially explained by the structural compensation (respective positions are marked by yellow arrows). Interestingly, near to these two additional insertions, it also has two unique functionally-considered mutations in the positions 25 and 27 respectively, that are not shared among other 18 Slovak isolates. Central Conserved Region (CCR), into which are the diagnostic primers targeted, is highlighted by red rectangle. Gibbs free energy for the most thermodynamically stable conformation was calculated via mFold software [37].
