# Supplementary material for: Analysis of Hop Stunt Viroid Diversity in Grapevine (Vitis vinifera L.) in Slovakia: Coexistence of Two Particular Genetic Groups
Source: Pathogens. 2023 Jan 28;12(2):205. doi: 10.3390/pathogens12020205 (PMC9965860; doi:10.3390/pathogens12020205)
Supplement: Supplementary file 1 [file pathogens-12-00205-s001.zip › Supplementary_Figure_S1_Alaxin_rev1.pptx]

## Slide 1
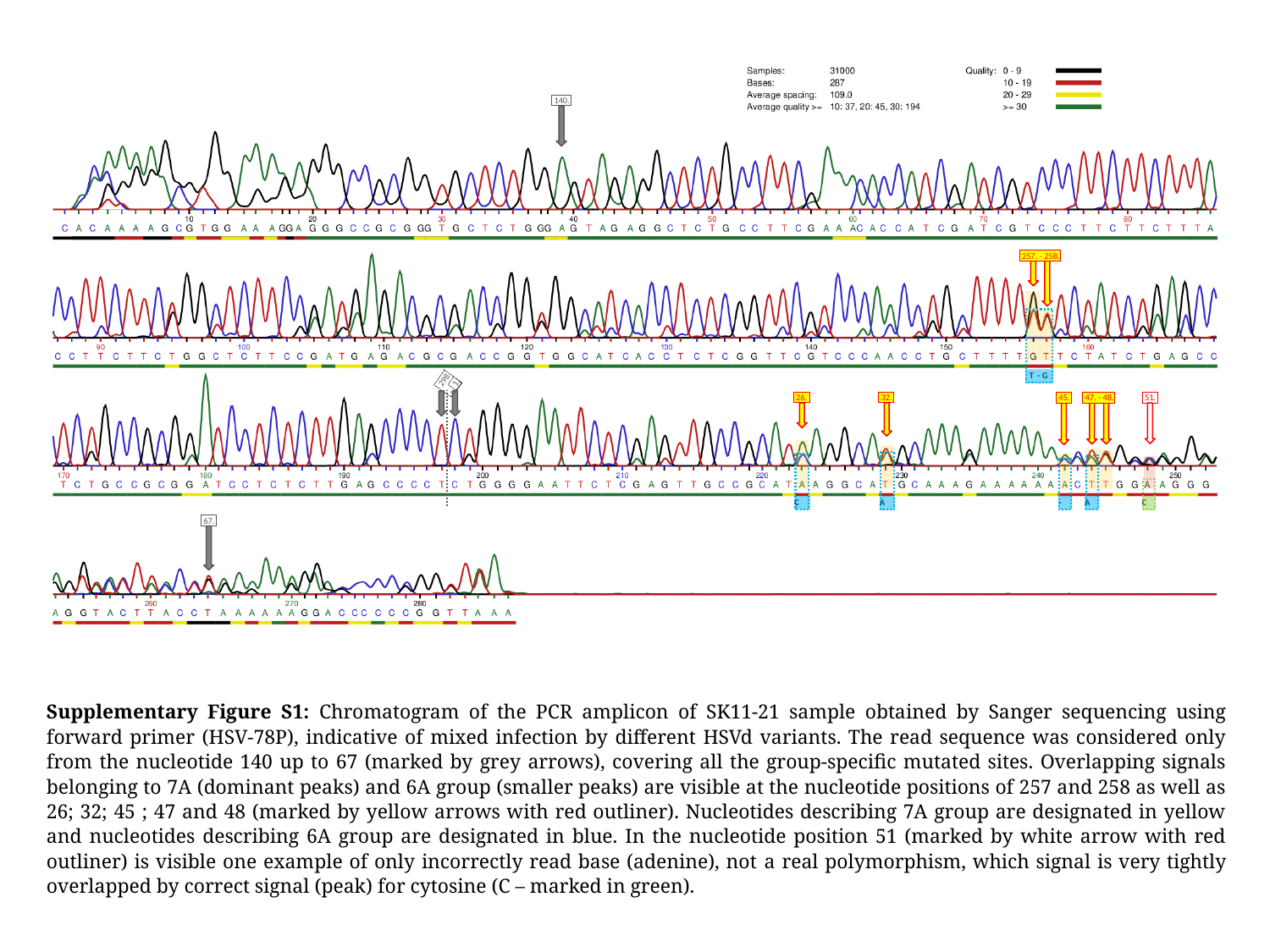

140.
257. - 258.
 T - G
298.
1.
26.
32.
45.
47. - 48.
51.
C
A
-
A
C
67.
Supplementary Figure S1: Chromatogram of the PCR amplicon of SK11-21 sample obtained by Sanger sequencing using forward primer (HSV-78P), indicative of mixed infection by different HSVd variants. The read sequence was considered only from the nucleotide 140 up to 67 (marked by grey arrows), covering all the group-specific mutated sites. Overlapping signals belonging to 7A (dominant peaks) and 6A group (smaller peaks) are visible at the nucleotide positions of 257 and 258 as well as 26; 32; 45 ; 47 and 48 (marked by yellow arrows with red outliner). Nucleotides describing 7A group are designated in yellow and nucleotides describing 6A group are designated in blue. In the nucleotide position 51 (marked by white arrow with red outliner) is visible one example of only incorrectly read base (adenine), not a real polymorphism, which signal is very tightly overlapped by correct signal (peak) for cytosine (C – marked in green).
